# Decreased expression of immune activator WARS1 and other pro-inflammatory mediators in type 1-like macrophages as an associated mode of action in response to fucoidan from marine brown algae

**DOI:** 10.3389/fimmu.2026.1868665

**Published:** 2026-09-03

**Authors:** Jasmin Ostermann, Timo Gemoll, Tabea Schnüttgen, Marie-Theres Tischhöfer, Kirstin Plötze-Martin, Jonas Fleckner, Karl-Ludwig Bruchhage, Susanne Alban, Ralph Pries

**Affiliations:** 1 Section for Translational Surgical Oncology and Biobanking, Department of Surgery, University of Luebeck, Luebeck, Kiel, Germany; 2 Department of Otorhinolaryngology, University of Luebeck, Luebeck, Kiel, Germany; 3 Pharmaceutical Institute, University of Kiel, Luebeck, Kiel, Germany

**Keywords:** fucoidan, inflammation, macrophages, proteomic analysis, *Saccharina latissima*, urokinase-type plasminogen activator receptor (uPAR), tryptophanyl-tRNA-synthetase 1 (WARS1)

## Abstract

**Background/aim:**

Macrophages are key regulators of innate and adaptive immune responses through antigen presentation and the production of inflammatory mediators and growth factors. Imbalances in the activation and inhibition of pro-inflammatory type 1-like macrophages (M1) and anti-inflammatory type 2-like macrophages (M2) are closely associated with various autoimmune and chronic inflammatory diseases, underscoring the need for targeted therapeutic approaches. Numerous studies have shown that sulfated polysaccharides from marine algae, especially fucoidans from brown algae, exhibit a wide range of anti-inflammatory activities. This study aims to investigate the anti-inflammatory mode of action of algae-derived fucoidans in monocyte-derived macrophages and to identify the key regulatory proteins and inflammatory mediators involved.

**Materials and methods:**

We applied data-independent mass spectrometry to comprehensively evaluate protein profiles of monocyte-derived M1/M2-like macrophages under the influence of sulfated polysaccharides from different algae species, namely fucoidans from the brown algae *Saccharina latissima*, *Fucus evanescens*, and *Laminaria digitata*, as well as the sulfated xylogalactan from the red alga *Delesseria sanguinea*. Furthermore, a series of gradually depolymerized fractions of the fucoidan from *Saccharina latissima* was investigated to elucidate the impact of molecular weight (MW) on protein expression levels and cytokine secretion patterns using membrane-based cytokine arrays and ELISA measurements.

**Results:**

Among the tested sulfated polysaccharides, especially the fucoidan from *Saccharina latissima* showed anti-inflammatory effects. They significantly reduced expression levels of pro-inflammatory tryptophanyl-tRNA synthetase (WARS1), the cytokines interferon gamma-induced protein 10 (IP-10, syn. CXCL10) and macrophage migration inhibitory factor (MIF), as well as urokinase-type plasminogen activator receptor (uPAR) in pro-inflammatory M1-like macrophages, whereby their effects turned out to be independent of the MW.

**Conclusion:**

Our study provides novel insights into the anti-inflammatory mode of action of fucoidan from *Saccharina latissimima* on monocyte-derived macrophages. Further *in vitro* and *in vivo* studies are required to better understand the underlying molecular pathways, the associated mechanism and to evaluate potential medical applications.

## Introduction

The first immune response in case of infection and inflammation is given by different myeloid cells of the innate immunity including monocytes, granulocytes, dendritic cells, and macrophages (1). Monocyte-derived macrophages are a particularly important part of the mononuclear phagocyte system of the innate immunity and essential regulators of both innate and adaptive immune responses via antigen presentation and the production of inflammatory mediators and growth factors (2, 3).

Macrophages have strong functional plasticity and are able to polarize into pro-inflammatory type 1-like macrophages (M1) and anti-inflammatory type 2-like macrophages (M2) depending on the microenvironmental situation (4, 5).

M1 macrophages are a major source of pro-inflammatory cytokines such as Interleukin (IL)‐1β, tumor necrosis factor (TNF)‐α, and IL‐6 and are important for immune surveillance (6). In contrast, M2 macrophages secrete anti-inflammatory cytokines and thereby control immune responses and promote angiogenesis and tissue remodeling (7–11). Imbalances in the activation and inhibition of M1/M2 macrophage subtypes are closely associated with various autoimmune and chronic inflammatory diseases, which are initiated by leukocyte migration into the inflamed lesion (12, 13). Recent findings underline the clinical importance of macrophage-mediated immune regulation of autoimmune diseases and chronic inflammation and the need for appropriate therapeutic strategies (1).

In this context, sulfated polysaccharides extracted from marine algae have attracted increasing attention as a promising anti-inflammatory therapeutic option, although the underlying molecular mechanisms remain poorly understood (14–19). In particular, fucose-containing sulfated polysaccharides, known as fucoidans, exhibit a wide range of anti-inflammatory and antiviral bioactivities and therefore appear promising for medical applications (20–23). Fucoidans from different brown algae species, such as *Fucus vesiculosus* and *Laminaria digitata*, were found to attenuate LPS-induced pro-inflammatory immune responses in both human and mouse macrophages, despite the considerable species-specific differences concerning the polarization pattern of monocyte-derived macrophages (24, 25).

However, comprehensive protein profiling of differentiation and polarization patterns of monocyte-derived M1/M2 macrophages with regard to the influence of marine fucoidans has not yet been carried out. In this context, proteomic analyses play a pivotal role in modern biomedical research by providing critical insights into biological processes. Since protein abundance, structure, and interaction networks shift in response to developmental, environmental, or disease-related factors, proteomic data offer a detailed picture of the actual phenotype of a cell or whole tissue, respectively. High-throughput mass spectrometry methods are characterized by high sensitivity and specificity, and enable comprehensive profiling of thousands of proteins at the same time, which is essential for identifying biomarkers, therapeutic targets or elucidating signaling pathways (26, 27).

The aim of the present study was to broaden our understanding of the anti-inflammatory modes of action of marine fucoidans on a cellular level and to identify key regulatory proteins involved. For this, we applied data-independent mass spectrometry to comprehensively evaluate protein profiles of monocyte-derived M1/M2-like polarized macrophages and their modulation by fucoidan from the marine brown algae *Saccharina latissima.* For comparison, fucoidans from *Fucus evanescens* and *Laminaria digitata*, a sulfated xylogalactan from the red alga *Delesseria sanguinea* as well as the low-molecular weight heparin (LMWH) tinzaparin and pentosan polysulfate (PPS), both approved for medical application, were included in the study.

Furthermore, we investigated the effect of gradually depolymerized fractions of the fucoidan from *Saccharina latissima* on protein expression and cytokine secretion patterns of M1-like macrophages.

## Materials and methods

### THP-1 culture conditions and macrophage polarization

Polarization of monocyte-derived M1/M2-like macrophages for proteomic analysis was performed using the human monocyte leukemia cell line THP-1 (Tohoku Hospital Pediatrics-1) to ensure a homogeneous genetic background and to minimize the variability in the macrophage phenotypes (28, 29).

Cell culture was performed in RPMI 1640 medium supplemented with 10% heat-inactivated fetal bovine serum (FBS), 1% sodium pyruvate and 1% streptomycin/penicillin at 37 °C and 5% CO_2_ under a humidified atmosphere. Cells were subcultured every three days when they reached a maximum density of 1x10^6^ cells/ml. To induce differentiation into monocyte-derived macrophages (MDMs), THP-1 cells were treated with 5 ng/ml phorbol-12-myristate-13-acetate (PMA) for 24 hours, and then further cultivated in fresh medium. After a four-day resting period, cells became adherent and developed a macrophage-like morphology corresponding to on-polarized (M0) macrophages. Next, MDMs were further differentiated into type 1-like macrophages (20 ng/ml IFNy, 10 ng/ml LPS) and type 2-like macrophages (20 ng/ml IL-4, 20 ng/ml IL-13) for 48 h.

### Test compounds

The extraction, purification, and structural characterization of the different sulfated algae polysaccharides have been carried out as previously described (30, 31). The native fucoidan from Saccharina latissima (MW 563 kDa) was gradually degraded by H_2_O_2_ treatment (32) resulting in fractions with MW down to 6 kDa (**Table 1**). The low-molecular weight heparin (LMWH) tinzaparin (CRS purchased from EDQM, Strasbourg, France) and pentosan polysulfate (PPS) (API obtained from bene pharmaChem GmbH & Co.KG, Geretsried, Germany) were used as reference substances. Preliminary viability assays were performed to ensure healthy living cells. The concentration of the test compounds in the cell culture experiments amounted to 50 µg/ml (33).

**Table 1:** List of test compounds.

Abbreviation	Type of sulfated polysaccharide	MW	DS
SL-Fuc-native	Fucoidan from Saccharina latissima (Kiel Fjord)	563 kDa	0.45
SL-Fuc-D1	Degraded fucoidan from SL-Fuc-native	270 kDa	0.47
SL-Fuc-D2	dito	124 kDa	0.38
SL-Fuc-D3	dito	88 kDa	0.45
SL-Fuc-D4	dito	61 kDa	0.47
SL-Fuc-D5	dito	20 kDa	0.46
SL-Fuc-D6	dito	6.1 kDa	0.54
FE-Fuc-1	Fucoidan from Fucus evanescens (Kiel Canal)*	181 kDa	0.53
FE-Fuc-2	Fucoidan from Fucus evanescens (Kiel Canal)*	543 kDa	0.44
SL-Fuc-Nor	Fucoidan from Saccharina latissima (Norway)	373 kDa	0.60
SL-Fuc-Nor-D	Degraded Fucoidan from SL-Fuc-Nor	48 kDa	0.63
LD-Fuc	Fucoidan from Laminaria digitata (Ireland)	616 kDa	0,39
DS-SXG	Sulfated xylogalactan from Delesseria sanguinea (Baltic Sea)	160 kDa	0.55
LMWH	Tinzaparin, a low-molecular weight heparin tinzparin	6.5 kDa	2.00
PPS	Pentosan polysulfate	5.4 kDa	1.99

MW = weight average molecular mass determined by SEC-MALS.

DS = degree of sulfation = sodium sulfate groups per monosaccharide.

*Harvest in different years.

### Protein extraction and digestion

Differentiated THP-1 cell extracts were prepared using the EasyPep Mini MS Sample Prep Kit (Thermo Fisher Scientific) according to the manufacturer’s instructions. Briefly, 5 million cells were lysed in lysis buffer to achieve a protein concentration of 1 mg/mL, and aliquots containing 50 μg of protein were treated with universal nuclease for 10 cycles of pipetting up and down until the viscosity decreased. Reduction and alkylation were achieved by adding the respective solutions and incubating the samples at 95 °C for 10 min. Once samples were cooled down, the trypsin/Lys-C protease mixture was added, and samples were digested at 37 °C for 3 h. Tryptic digestion was stopped using the Digestion Stop Solution. Peptide Cleanup columns were cleared from the liquid by centrifugation and placed onto 2 mL microcentrifuge tubes. Sample mixtures were transferred into dry Peptide Cleanup columns. Two consecutive rounds of centrifugation and washing were performed. The columns were transferred to 2 mL microcentrifuge tubes, and peptides were eluted by adding the elution solution and centrifuging at 1,500*g* for 2 min. Samples were dried using a vacuum centrifuge and resuspended in 0.1% formic acid for MS analysis. The protein concentration after cell lysis and after protein precipitation was determined using the DC Protein Assay (Bio-Rad, Hercules, CA, USA) according to the manufacturer’s guidelines.

### LC-ESI-MS/MS analysis

Aliquots of 200 ng of the purified peptides were loaded onto C18 EvoTip disposable trap columns (EV 1137, 15 cm x 150 µm, 1.5 µm). Subsequently, peptides were chromatographically separated using an EvoSep One (EvoSep, Denmark) with the following parameters: 15 SPD (samples per day) method, 88 min gradient, 220 nL/min. A timsTOF HT flex mass spectrometer (Bruker, Germany) was used to measure the peptides in a label-free data-independent acquisition (DIA) mode.

### Protein quantification and bioinformatics analysis

The DIA-NN software and the diann R Package were used for protein identification and quantification. Unique proteotypic peptides with a false discovery rate (FDR) below 0.01 were included in the analysis. Data preprocessing was carried out using the R packages DEP and missForest. First, proteins were filtered for completeness using a fraction filter (cut-off 70%). Second, median normalization and log_2_ transformation were applied. Third, missing values were imputed using randomForest-based imputation.

An unsupervised principal component analysis (PCA) was performed to assess sample clustering in the dataset. Differential expression analyses were carried out using the limma (Linear Models for Microarray Data) R Package. P-values were adjusted for multiple testing using the Benjamini-Hochberg (BH) procedure. Proteins with FDR < 0.05 and |log_2_FC| > 1 were considered significantly differentially expressed.

To visualize WARS1 protein expression across all differentiation conditions, boxplots were created. The significance values shown represent the adjusted p-values calculated in limma. A gene set enrichment analysis (GSEA) was performed using MSigDB Hallmark (R packages clusterProfiler, msigdbr) with a gene set size range of 5 to 500 and an BH-adjusted p-value cutoff of 0.25. Normalized Enrichment Scores (NES) were calculated to indicate the direction of enrichment. For all significantly enriched pathways, the core (leading-edge) enrichment genes were extracted from the GSEA output. Pathways in which WARS1 was identified as part of the leading-edge genes were selected for visualization. For these, a running enrichment score was calculated according to the algorithm of Subramanian et al. (2005) (34). A protein-protein interaction (PPI) network was constructed based on STRING database interactions (R package STRINGdb) with a combined confidence score threshold of ≥ 400. First-degree interaction partners of WARS1 were identified from the STRING database and intersected with the proteomics data. Subsequently, Pearson correlation coefficients between WARS1 and each interacting partner were calculated for comparing M1-like macrophages to those in response to fucoidan. The PPI network for the top-correlated interactions of WARS1 was visualized (R packages igraph, tidygraph) using a Fruchterman-Reingold (FR) layout, with Louvain clustering applied to identify the network modules.

### Western hybridization analysis

Cells were washed with ice-cold PBS and protein extraction was carried out using reagents purchased from Thermo Fisher Scientific (Waltham, MA, USA) according to the manufacturer’s protocol. Protein concentration was determined using Bio-Rad DC Protein Assay (Bio-Rad, Hercules, CA, USA). Proteins were separated by SDS-PAGE and transferred onto polyvinylidene difluoride membrane (PVDF, Merck Millipore, Burlington, MA, USA) by semi-dry electroblotting. The blots were incubated with primary antibodies for 24 h and with horseradish peroxidase (HRP)-conjugated secondary antibodies (Cat: 7074P2; Cell Signaling Technology, Leiden, The Netherlands) in a concentration of 1:1000 for 1 h and finally detected by electrochemiluminescence (ECL, Bio-Rad, Hercules, CA, USA).

Antibodies used were anti-WARS1 (Thermo Fisher Scientific, Waltham, MA, USA; Cat: PA5-29102) and anti-GAPDH (Cell Signaling Technology, Leiden, The Netherlands; Cat: 2118). Molecular weight was calculated using the Page Ruler plus™ protein ladder (Thermo Fisher Scientific, Waltham, MA, USA; Cat: 26619).

### Cytokine analysis

Cell culture supernatants were collected and instantly frozen with liquid nitrogen and preserved at -80 °C. Analyses of cytokines and chemokines were performed using membrane-based Proteome Profiler™ Human XL cytokine arrays (R&D Systems, Minneapolis, MN, USA) as recommended by the supplier. Expression was visualized using an enhanced chemiluminescence detection kit (R&D Systems, Minneapolis, USA). Semiquantitative analysis was performed by measuring dot density using an iBright CL 1000 biomolecular imager (Invitrogen, Carlsbad, CA, USA). Secretion levels of urokinase-type plasminogen activator receptor (uPAR) by monocyte-derived M1-like macrophages were determined by enzyme-linked immunosorbent assays (ELISA) according to the manufacturer’s protocols (R&D Systems, Minneapolis, MN, USA).

### Statistical analyses

Statistical analyses were performed with GraphPad Prism Version 7.0f. Means and standard errors of the mean (SEM) are presented. The differences between groups were determined after testing for Gaussian distribution (normality tests) and applying parametric (Student`s t-test), or non-parametric 1-way ANOVA with Bonferroni *post hoc* test. Correlations between parameters were assessed using multivariate regression with Pearson correlation coefficients. p < 0.05 (*), p < 0.01 (**), and p < 0.001 (***). Additional statistical details such as sample size are given in the respective figure legends, when appropriate.

## Results

### Proteomic analysis of monocyte derived macrophages

**Figure 1:**
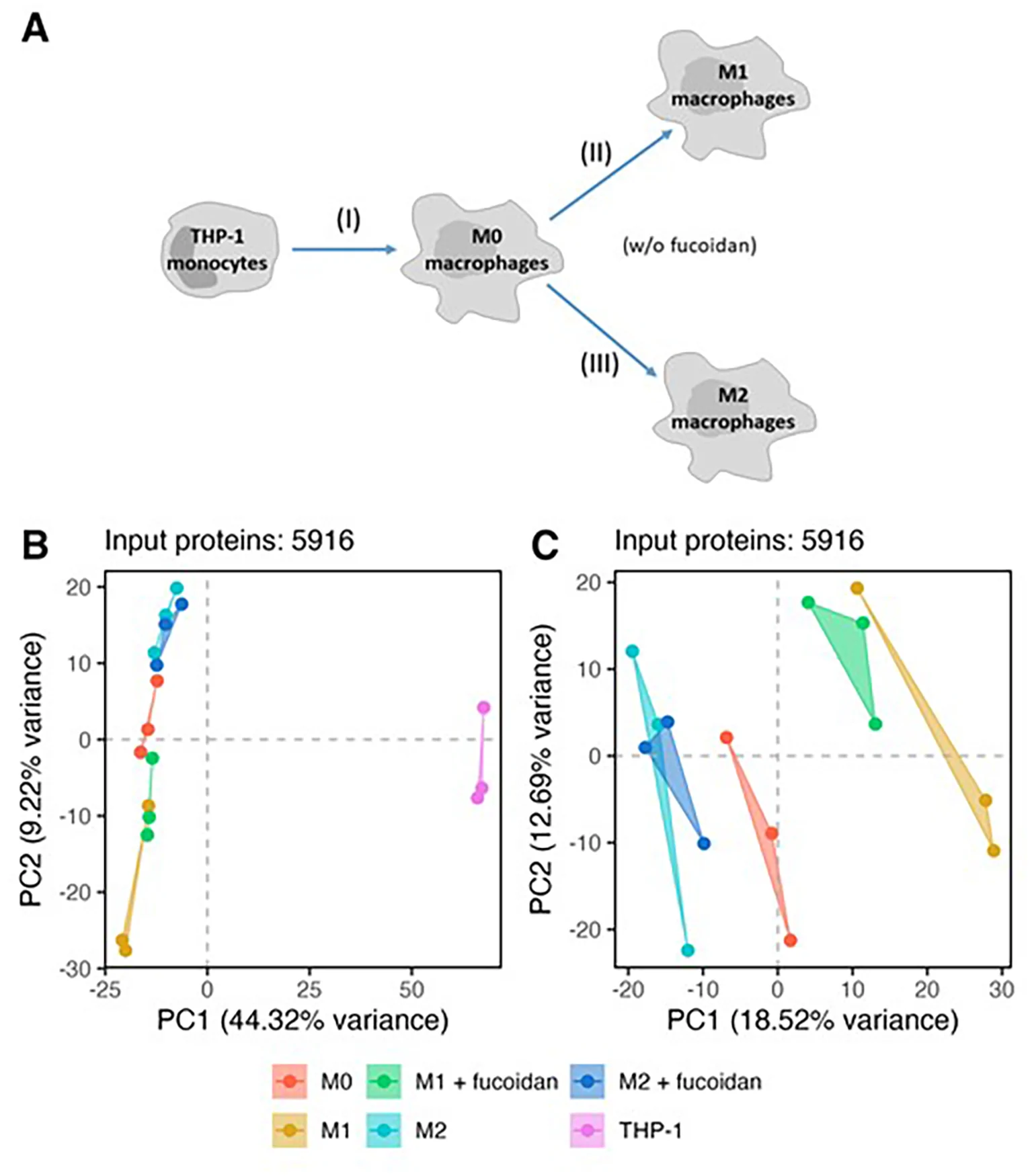
Proteomic analysis of monocyte-derived macrophages. **(A)** To induce differentiation into monocyte-derived macrophages, THP-1 cells were (I) treated with phorbol-12-myristate-13-acetate (PMA) to receive M0 macrophages, which were further differentiated into (II) type 1-like macrophages (M1) and (III) type 2-like macrophages (M2) in the absence or presence of the native fucoidan (w/o fucoidan) from *Saccharina latissima* (50 µg/ml). **(B)** Principal component analysis (PCA) of quantitative proteomics (n = 3) of the different analyzed groups (THP-1, M0 macrophages, M1-like macrophages, M2-like macrophages, M1-like macrophages + fucoidan, M2-like macrophages + fucoidan). **(C)** PCA of quantitative proteomics (n = 3) of the different analyzed groups without THP-1.

Non-adherent THP-1 monocytes were differentiated into adherent non-polarized M0 macrophages and then further polarized into M1/M2-like macrophages in the presence and absence of the native fucoidan from *Saccharina latissima* (50 µg/ml) (**Figure 1A**).

Protein extracts were obtained from three independent experimental approaches of macrophage polarization and analyzed by quantitative proteomics. A total of 6,609 proteins were identified, of which 5,916 were used for data analysis after data preprocessing. Clustering of the distinct subgroups via principal component analysis (PCA) revealed a strong clustering within the subset replicates (n = 3) and indicated that THP-1 monocytes and M1-like and M2-like macrophages were clearly distributed into different quadrants (**Figure 1B**).

Of note, M0 and M2-like macrophages were clustered in the same quadrant, indicating similarities in the underlying protein expression profiles. In contrast, M1-like macrophages were clustered in another quadrant and additionally displayed larger protein shifts in response to fucoidan (**Figures 1B, C**).

**Figure 2:**
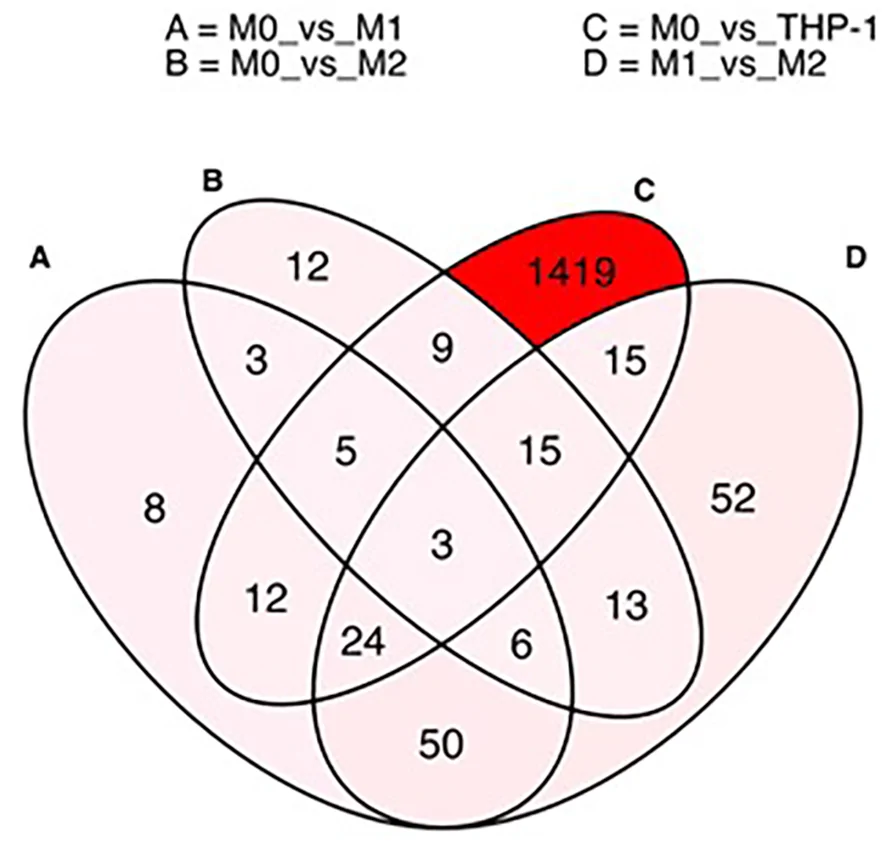
Venn diagram of quantifiable proteins among the analyzed subgroups of monocyte-derived macrophages.

Venn diagram illustration of quantifiable proteins among the analyzed subgroups of monocyte-derived macrophages revealed the most pronounced alteration of protein expression profile during the conversion of non-adherent THP-1 monocytes to adherent M0 macrophages, while significantly fewer alterations of protein expression levels were observed among the M0/M1-like/M2-like macrophage subtypes (**Figure 2**).

### Protein profiles in the course of monocyte-derived macrophage differentiation

**Figure 3:**
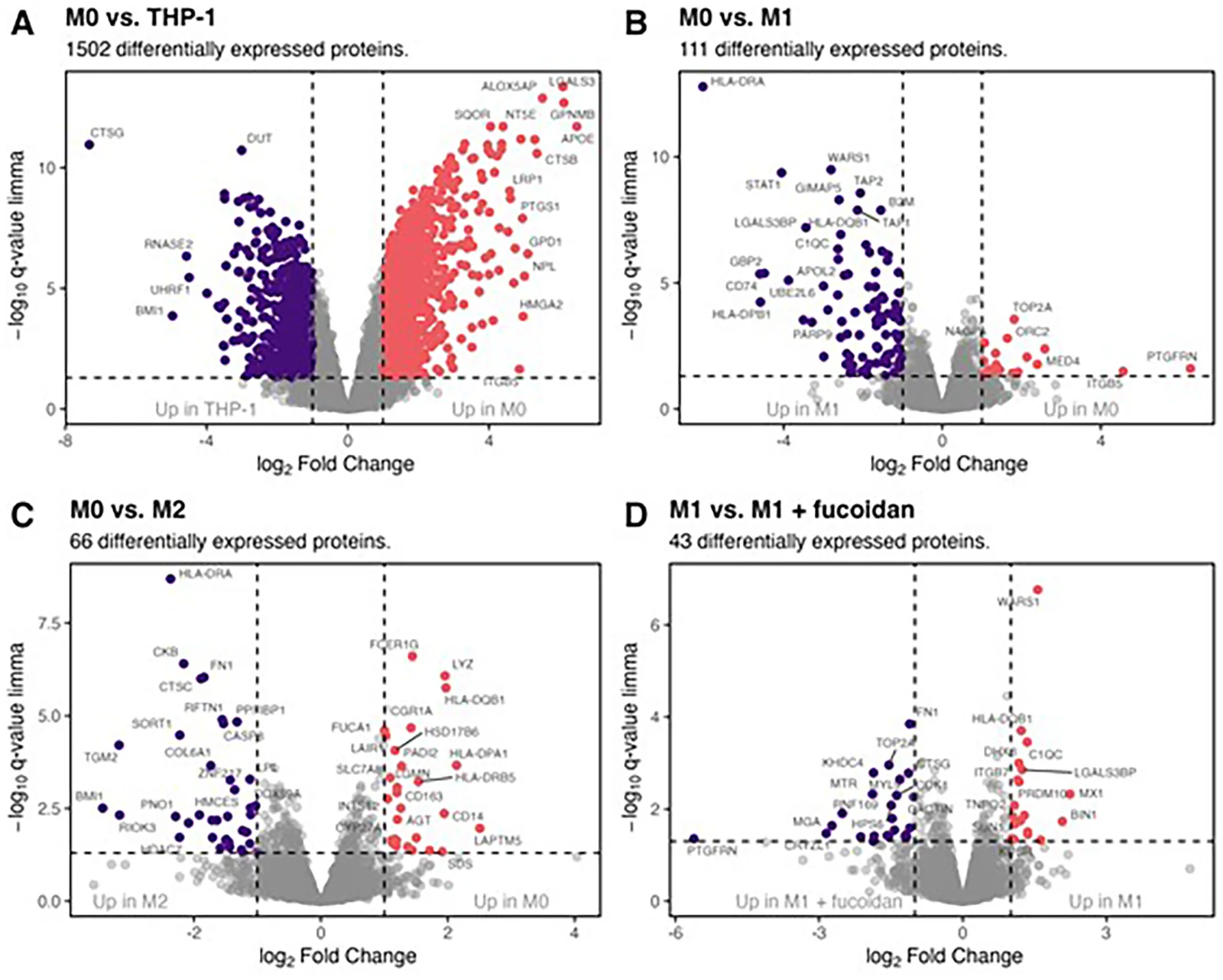
Volcano plots depicting the variances in protein expression between the different subgroups of differentiation of monocyte derived macrophages [**(A)** M0 vs. THP-1, **(B)** M0 vs. M1, **(C)** M0 vs. M2] and the influence of the native fucoidan from *Saccharina latissima* [**(D)** M1 vs. M1 + fucoidan]. Data revealed **(B)** strongly increased levels of pro-inflammatory tryptophanyl-tRNA synthetase (WARS1) in M1-like macrophages compared to M0 macrophages as well as **(D)** strongly decreased WARS1 levels in M1-like macrophages in response to the fucoidan from *Saccharina lattissima* (50 µg/ml).

Quantitative proteomics of the conversion of THP-1 monocytes to M0 macrophages identified numerous macrophage-related proteins significantly induced by the conversion, including apolipoprotein E (APOE), leukocyte‐specific protein 1 (LSP1), and matrix metalloproteinase 9 (MMP9) (**Figure 3A**). Furthermore, data revealed increased protein levels of arachidonate 5-lipoxygenase activating protein (ALOX5AP) and heme oxygenase 1 (HO-1) in M0 macrophages compared to THP-1 monocytes, both of which are also known to be associated with a M2-like macrophage polarization (35, 36) (**Figure 3A**).

These findings corroborate the PCA clustering of M0 and M2-like macrophages in the same quadrant due to protein profile similarities (**Figures 1B, C**). As expected, proteomic analysis of the M0 to M1-like macrophage polarization revealed strongly increased expression of established M1 marker proteins such as human leukocyte antigen DR (HLA-DR), activator of transcription 1 (STAT1), and CD74 (**Figure 3B**). Polarization of M0 to M2-like macrophages resulted in increased expression of the M2 macrophage marker CD209, but significantly lower levels of distinct HLA-DR proteins compared to M1-like macrophages (**Figure 3C**).

Of note, increased protein levels of pro-inflammatory tryptophanyl-tRNA synthetase (WARS1) were identified in M1-like macrophages (**Figure 3B**), which were significantly reduced in response to the fucoidan from *Saccharina latissima* (**Figure 3D**).

**Figure 4:**
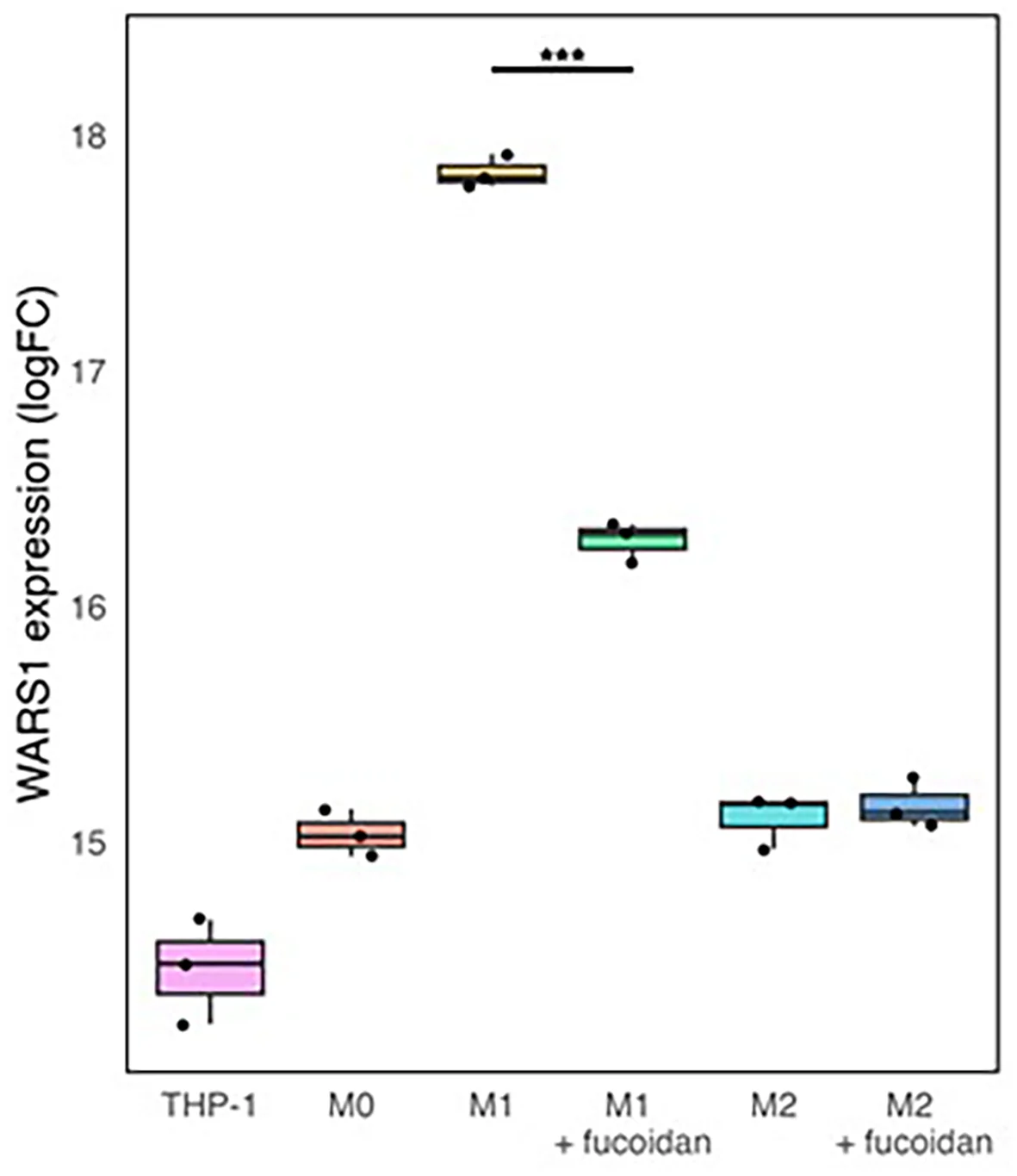
WARS1 protein profiles of non-adherent THP-1 monocytes and adherent M0/M1/M2 macrophage subtypes in the with regards to fucoidan. WARS1 expression boxplots indicate significantly increased expression of WARS1 in M1-like macrophages and a significant reduction in response to native fucoidan from *Saccharina latissimi* (50 µg/ml; n = 3). Asterisks indicate statistically significant adjusted p-values calculated in limma. p < 0.05 (*), p < 0.01 (**), p < 0.001 (***).

These findings are clearly illustrated by the corresponding WARS1 expression boxplots (**Figure 4**).

**Figure 5:**
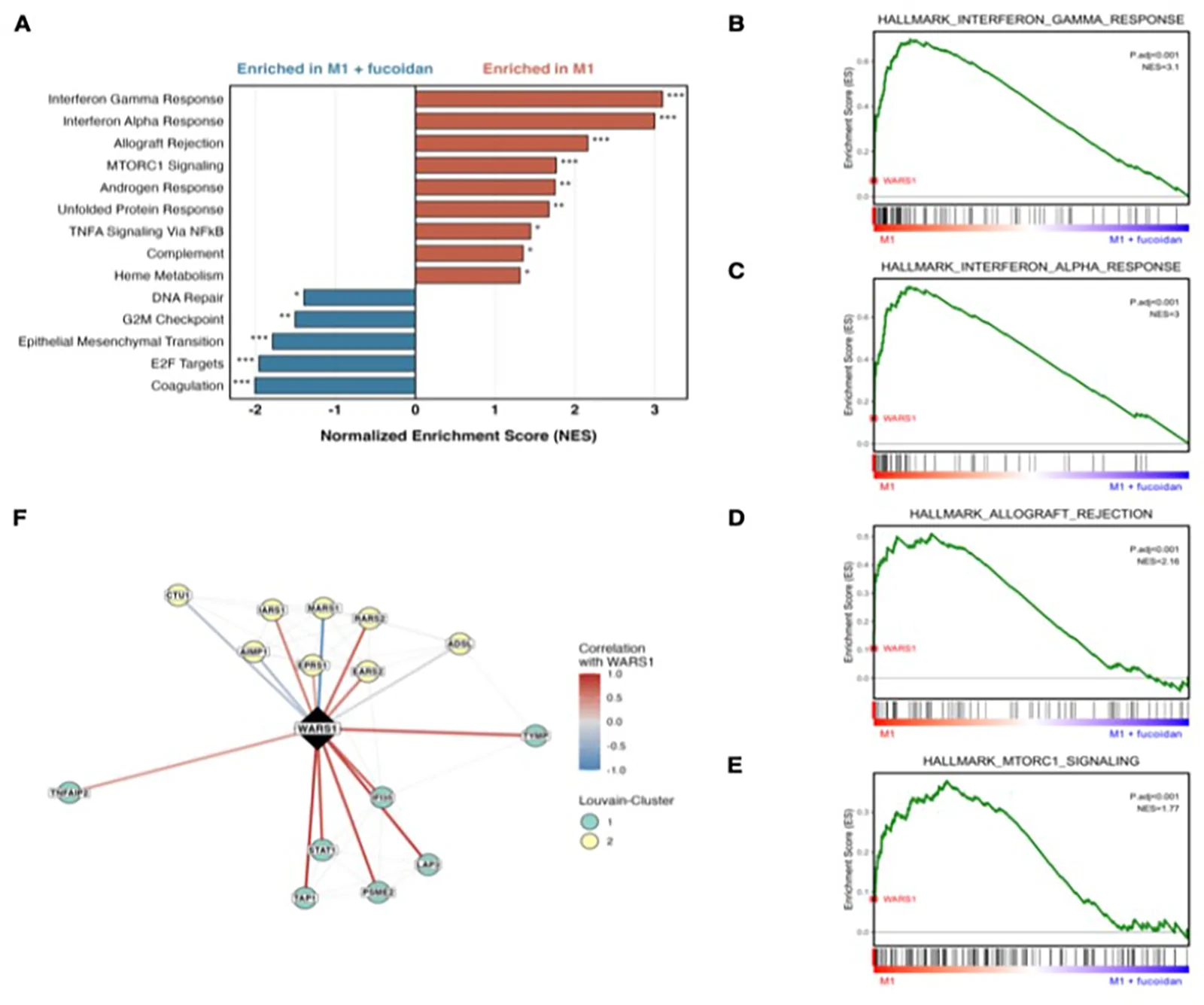
Attenuated inflammatory signaling through fucoidan treatment in M1-like macrophages. **(A)** GSEA of MSigDB Hallmark gene set comparing M1-like macrophages with those treated with fucoidan. Asterisks indicate BH-adjusted p-values with p < 0.25 (*), p < 0.05 (**), p < 0.01 (***). **(B–E)** Enrichment plots for significant pathways with WARS1 in the leading edge: Interferon Gamma Response **(B)**, Interferon Alpha Response **(C)**, Allograft Rejection **(D)**, MTORC1 Signaling **(E)**. **(F)** WARS1-centered PPI network. Nodes are colored according to the Louvain community assignment. Edges are colored to indicate Pearson correlation between WARS1 and its interacting proteins when comparing M1-like macrophages to those in response to fucoidan. Red and blue colors represent positive and negative correlation.

GSEA using the MSigDB Hallmark gene set revealed significant downregulation of pro-inflammatory signaling pathways, such as the Interferon Gamma Response, in M1-like macrophages following fucoidan treatment (**Figure 5A**). Four of the significant pathways included WARS1 among their leading-edge genes: Interferon Gamma Response (**Figure 5B**), Interferon Alpha Response (**Figure 5C**), Allograft Rejection (**Figure 5D**), and MTORC1 Signaling (**Figure 5E**). Across all four pathways, WARS1 was strongly associated with M1-like macrophage phenotype. These findings suggest that the fucoidan-induced reduction of WARS1 is part of a broader inflammatory event.

A PPI network analysis centered on WARS1 (**Figure 5F**) also identified two distinct network modules. Louvain Cluster 1 was primarily associated with interferon response, including proteins such as STAT1 and IFI35, and showed a significant positive correlation with WARS1. In contrast, Louvain Cluster 2 was associated with translation and tRNA aminoacylation, including proteins such as IARS1, MARS1, and RARS2. This cluster showed heterogeneous correlation with WARS1.

### Reduction of WARS1 expression by sulfated polysaccharides

**Figure 6:**
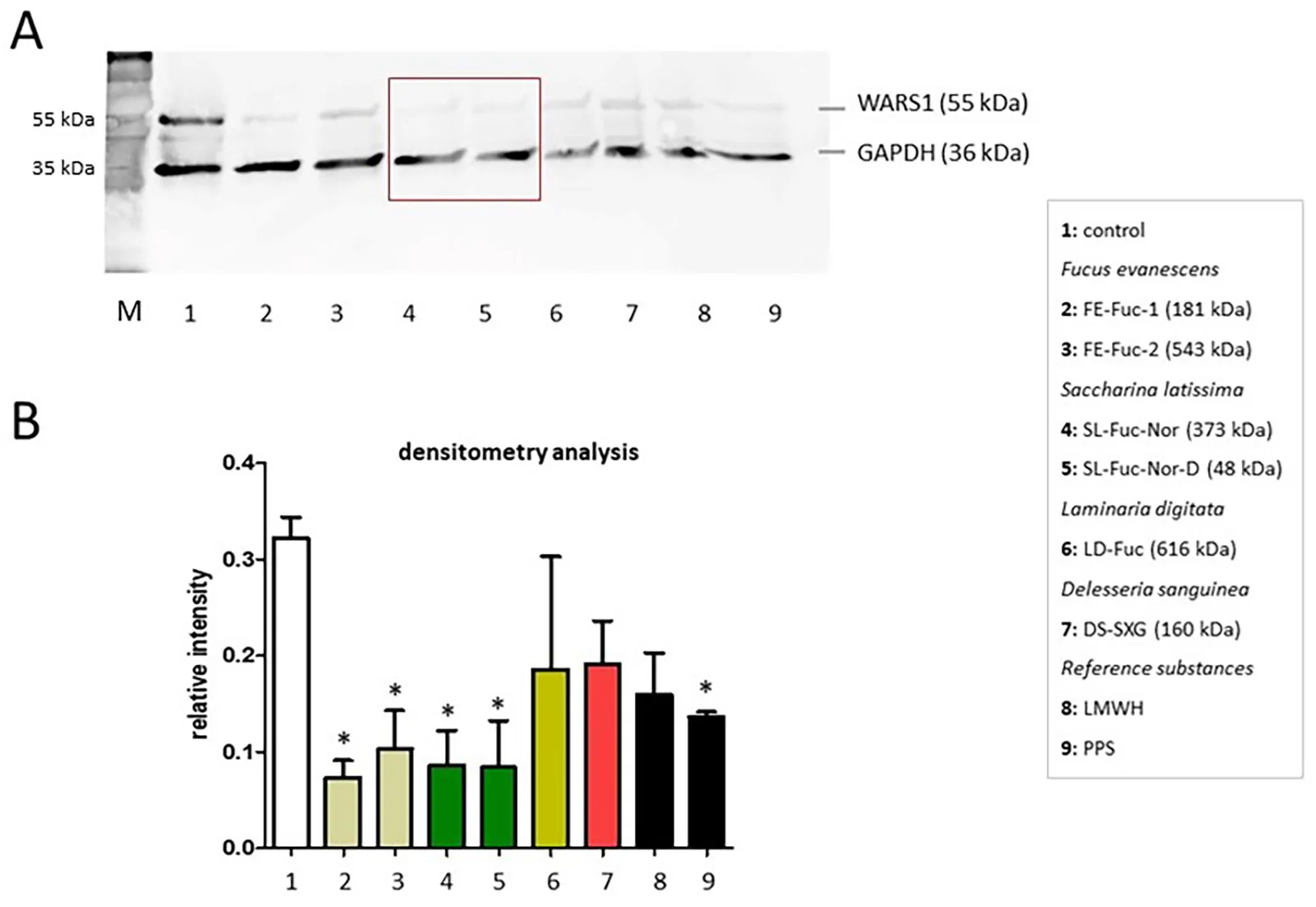
WARS1 expression in M1-like macrophages. **(A)** Representative WARS1 western blot images from M1 polarized macrophages in the absence (1: control) and presence of sulfated polysaccharides extracted from various algae as well as the reference substances LMWH tinzaparin and PPS (50µg/ml) (2–9). The red rectangle marks the protein bands of *Saccharina latissima* which were used for further experiments **(B)** Semiquantitative analysis was performed by measuring the density of the bands of three independent experiments and revealed differentially decreased WARS1 expression levels in response to different sulfated polysaccharides. WARS1 band density was normalized to the GAPDH loading control. M: Molecular weight marker with size annotations.

WARS1 acts as an innate immune activator involved in the regulation of local and systemic inflammation (37). Therefore, further investigations were performed To corroborate the observed reduced expression of WARS1 by the fucoidan from *Saccharina latissima*, six further sulfated polysaccharides, as well as LMWH tinzaparin and PPS as prime examples of biologically active sulfated glycans were investigated concerning such an effect. The algae polysaccharides included two fucoidan batches from *Fucus evanescens*, another native and a degraded fucoidan batch from *Saccharina latissima* (different origin), fucoidan from *Laminaria digitata*, and a sulfated xylogalactan from the red alga *Delesseria sanguinea*. In Western hybridization experiments all tested sulfated polysaccharides clearly reduced WARS1 expression compared to the control. Strongest effects were observed in response to the fucoidans from *Fucus evanescens* and *Saccharina latissima*, even when compared to LMWH tinzaparin and PPS (**Figure 6**).

**Figure 7:**
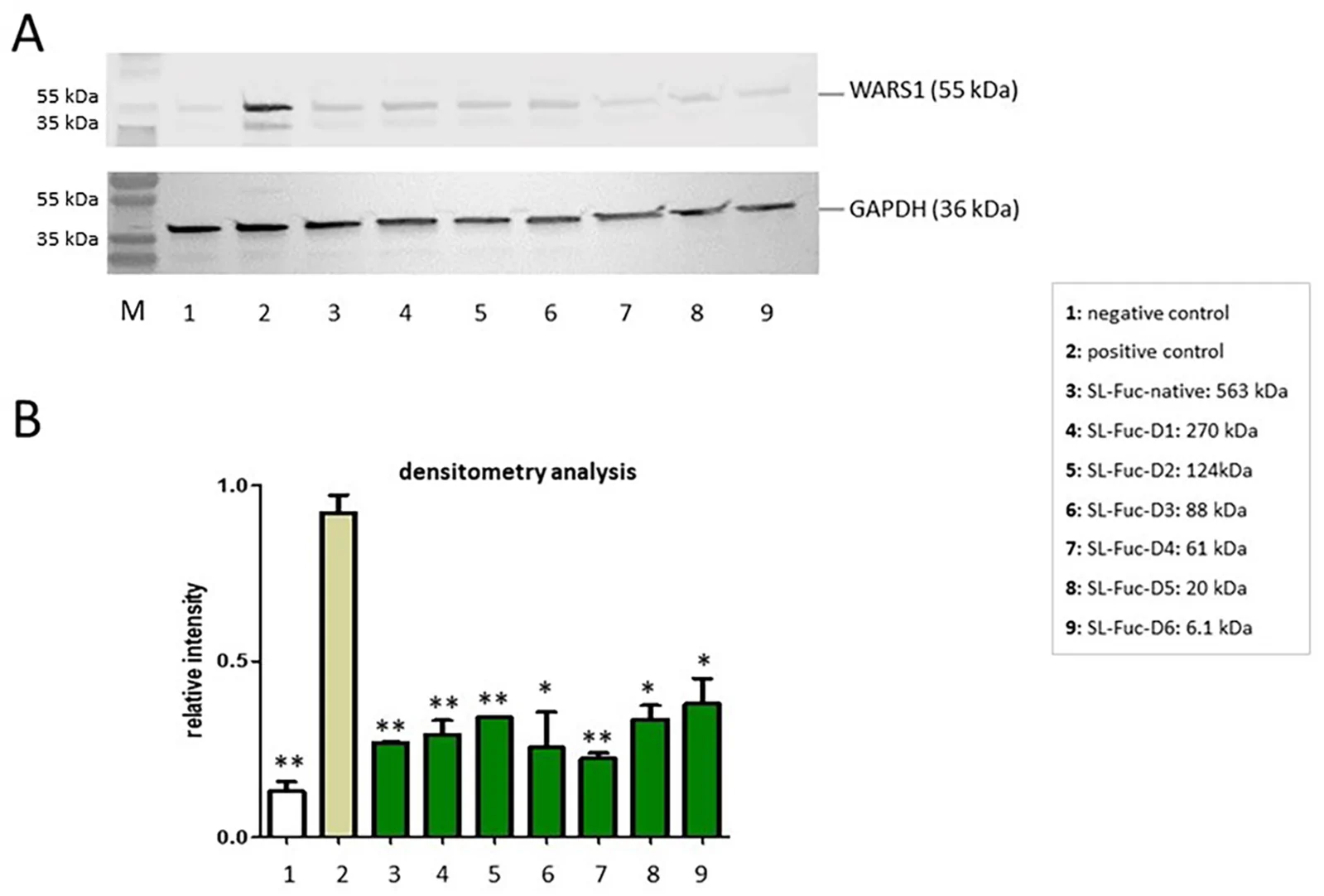
WARS1 expression in M1-like macrophages in response to native fucoidan from *Sacccharina latissima* and six degraded fractions with gradually reduced MW (50µg/ml; SL-Fuc-native: 563 kDa; SL-Fuc-D1: 270 kDa; SL-Fuc-D2: 124kDa; SL-Fuc-D3: 88 kDa; SL-Fuc-D4: 61 kDa; SL-Fuc-D5: 20 kDa; SL-Fuc-D6: 6.1 kDa) compared to the internal controls (1: negative ‘medium’ control; 2: positive M1 ‘IFN-y’ control). **(A)** Representative WARS1 western blot images from M1-polarized macrophages in response to the indicated culture conditions. **(B)** Semiquantitative analysis was performed by measuring the density of the bands of three independent experiments and revealed decreased WARS1 expression levels in response to the different fucoidans. WARS1 band density was normalized to the GAPDH loading control. M: Molecular weight marker with size annotations.

Earlier publications showed that the pharmacological activities of fucoidans are dependent on their MW and decrease with decreasing MW (32, 33, 38). On the other hand, the reduction of the high MW of native fucoidans improves their biopharmaceutical characteristics. Therefore, we investigated the effect of the MW of fucoidan on the WARS1 expression in M1-like macrophages. For this, the native fucoidan from *Saccharina latissima* (MW 563 kDa) was gradually depolymerized resulting in six fractions with MW ranging from 270 kDa down to 6 kDa (**Figure 7**).

Western hybridization experiments revealed that all the degraded fucoidan fractions significantly reduced WARS1 expression by more than 60% compared to the WARS1 baseline expression in M1-polarized macrophages and their effect did not significantly differ from that of the native fucoidan (**Figure 7**).

### Cytokine secretion in response to fucoidans

**Figure 8:**
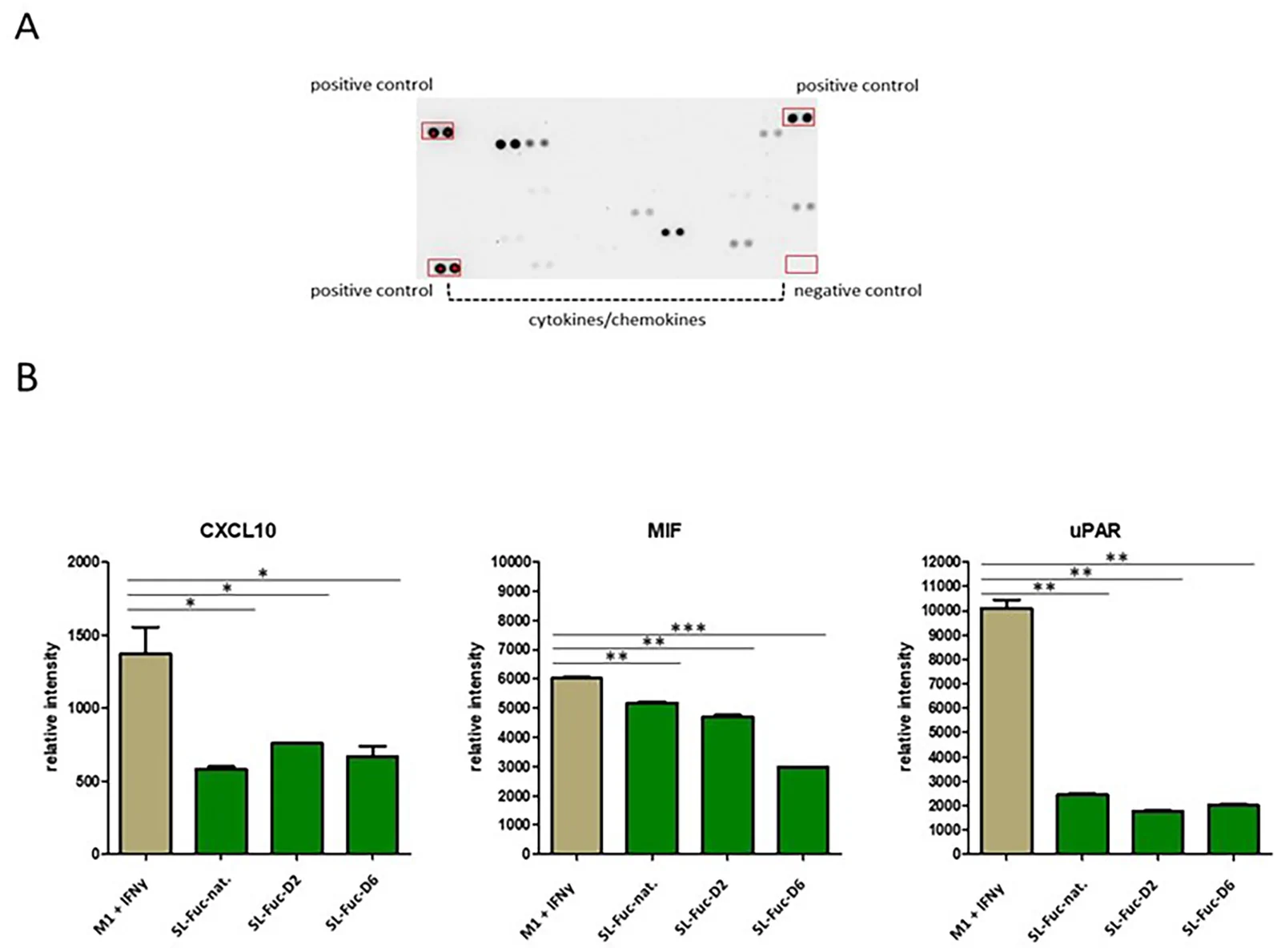
Cytokine secretion pattern of M1-like macrophages in response to native fucoidan from *Saccharina latissima* and the degraded fractions SL-Fuc-D2 (124 kDa) and SL-Fuc-D6 (6.1 kDa) (50 µg/ml; n = 3). **(A)** Example image of a membrane-based cytokine array of cell culture supernatants illustrating the membrane organization of positive and negative controls and cytokine dots. **(B)** Semiquantitative analysis was performed by measuring the density of the dots and revealed decreased secretion levels of cytokines interferon gamma-induced protein 10 (IP-10/CXCL10), macrophage migration inhibitory factor (MIF), and urokinase-type plasminogen activator receptor (uPAR) in response to the three tested fucoidans (M1 + IFNγ). *p < 0.05; **p < 0.01; ***p < 0.001.

Since fucoidan down-regulated the expression of the native immune activator WARS1, the question arose whether this results in a reduced secretion of proinflammatory cytokines by M1-like macrophages. Therefore, secretion patterns of 105 different cytokines and chemokines were evaluated using membrane-based human cytokine arrays. The cell culture supernatants of M1-like macrophages were analyzed in response to the absence and presence of native fucoidan from *Saccharina latissimi*, as well as two degraded fractions (SL-Fuc-native (563 kDa); D2 (124 kDa); D6 (6 kDa)) (**Figure 8**).

In particular, semiquantitative analysis demonstrated significantly decreased secretion of the cytokines interferon gamma-induced protein 10 (IP-10/CXCL10), macrophage migration inhibitory factor (MIF) and urokinase-type plasminogen activator receptor (uPAR) by M1-like macrophages in response to the three fucoidans from *Saccharina latissima* (**Figure 8**), whereby the effects on the MIF release event slightly improved with decreasing MW.

Among these, uPAR is of particular interest, since it has recently been recognized as a key immune regulator in different inflammatory diseases. Therefore, further ELISA measurements were performed to comprehensively quantify the influence of the native fucoidan from *Saccharina latissima* and the six degraded fractions on the uPAR secretion pattern by M1-like macrophages (**Figure 8**).

**Figure 9:**
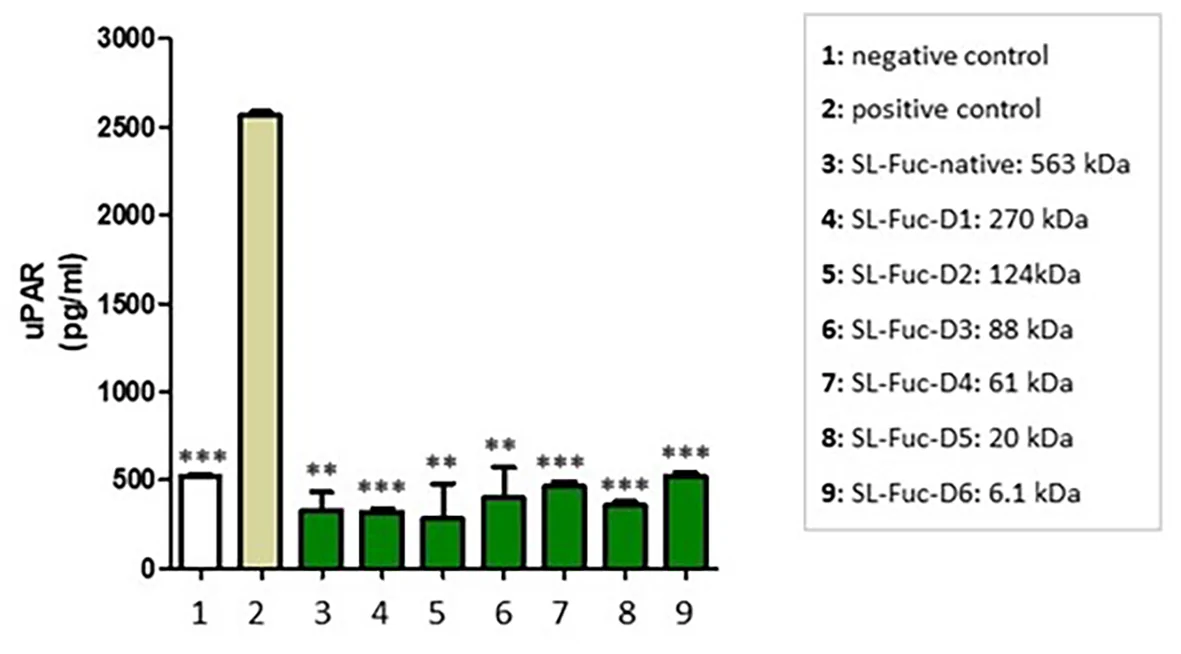
ELISA measurements of urokinase-type plasminogen activator receptor (uPAR) by M1-like macrophages in response to native fucoidan from *Sacccharina latissima* and six degraded fractions with gradually reduced MW (50µg/ml; n = 3; SL-Fuc-native: 563 kDa; SL-Fuc-D1: 270 kDa; SL-Fuc-D2: 124kDa; SL-Fuc-D3: 88 kDa; SL-Fuc-D4: 61 kDa; SL-Fuc-D5: 20 kDa; SL-Fuc-D6: 6.1 kDa) compared to the baseline secretion from expression in M1-polarized macrophages (1: negative ‘medium’ control; 2: positive M1 ‘IFN-y’ control). **p < 0.01; ***p < 0.001.

The ELISA results confirmed a highly significant decrease in uPAR secretion in response to the fucoidans compared to the baseline uPAR secretion level in M1-like polarized macrophages as well as the absence of any MW-dependent effect (**Figure 9**).

## Discussion

Macrophage differentiation is a very dynamic and versatile process and essential for the regulation of immune responses in infectious and inflammatory diseases (39). In this context, pro-inflammatory M1-like macrophages and anti-inflammatory M2-like macrophages are essential to balance both the rapid initiation as well as the required termination of immune defenses in human health (40). Different monocytic cell lines have been established for *in vitro* investigations on macrophage biology and polarization, although they are certainly simplified experimental modes of the *in vivo* situation and display certain differences compared to primary human macrophages (41–43).

In this study, proteomic analysis confirmed that the THP-1 monocytes were successfully differentiated into M0, M1-like, and M2-like macrophages. Differentiation of THP-1 monocytes to non-polarized M0 macrophages resulted in increased expression of proteins (e. g. apolipoprotein E (APOE), leukocyte‐specific protein 1 (LSP1), or matrix metalloproteinase 9 (MMP9)) known to be typical for macrophages. It has recently been shown that APOE is involved in the regulation of dectin-1-dependent development of monocyte-derived alveolar macrophages and is associated with elevated phagocytic activity (44). In accordance with the increased expression of LSP1 after the differentiation of non-adherent THP-1 monocytes to adherent M0 macrophages, the LSP1‐myosin1e bimolecular complex was identified as an essential regulator of adhesion, actin cytoskeleton remodeling, and migration (45). Moreover, MMP9 is known to be only scarcely expressed by circulating monocytes, whereas macrophage differentiation and increased migration are associated with elevated MMP9 levels (46, 47).

PMA-induced non-polarized M0 macrophages revealed furthermore increased abundances of arachidonate 5-lipoxygenase activating protein (ALOX5AP) and heme oxygenase 1 (HO-1) compared to THP-1 monocytes, both of which are also associated with an M2-like macrophage polarization (35, 36). These findings corroborate that M0 and M2-like macrophages were clustered in the same quadrant due to protein profile similarities.

Proteomic analysis of the M0 to M1-like macrophage polarization revealed strongly increased expression levels of M1 markers such as human leukocyte antigen DR (HLA-DR), activator of transcription 1 (STAT1) and CD74, whereas the M0 to M2-like macrophage polarization revealed increased expression levels of the established M2-like macrophage marker CD209 (48) and significantly lower levels of HLA-DR proteins. The HLA-DR is known to be abundantly expressed on human monocytes and macrophages and required for antigen presentation to CD4-positive T-lymphocytes, whereas elevated HLA-DR is a characteristic protein marker for M1-like macrophages (9). Signaling by the IFN-γ receptor activates the STAT1 pathway to induce the expression of classical interferon-stimulated pro-inflammatory genes (49). M1 macrophage marker CD74 is a type-II transmembrane glycoprotein that functions as a chaperone in the transport of MHC II molecules within the process of antigen presentation and is a receptor protein for the macrophage-migration inhibitory factor (MIF) (50, 51).

Concerning fucoidan, the proteomic results demonstrated that it modulated the differentiation to both M1- and M2-like macrophages, but more strongly affected the differentiation to pro-inflammatory M1- than to anti-inflammatory M2-like macrophages. Especially noteworthy, was the reduced expression of Tryptophanyl-tRNA synthetase (WARS1) in M1-polarized macrophages. This effect was confirmed by investigating a series of further sulfated polysaccharides. Among the tested series, the fucoidans from *Saccharina latissima* and *Fucus evanescens* turned out to be most effective.

This is thought to be an important result, since WARS1 is not only known to catalyze the ligation of tryptophan to its corresponding tRNA molecules within the process of translation (52), but also acts as an innate immune activator *in vivo* and is involved in the regulation of local and systemic inflammation upon infection (37). In response to invading pathogens, WARS1 is rapidly secreted from monocytes and macrophages and stimulates the production of various pro-inflammatory mediators such as TNF-α, IL-6, IL-8, MIP-1α, IFN-α, and IFN-β via toll-like receptor (TLR) 2 and TLR4 (53–55). It has been shown in hyperinflammatory sepsis that elevated plasma WARS1 levels stratified the early death of critically ill sepsis patients in correlation with increased levels of pro-inflammatory cytokines and absolute numbers of neutrophils and monocytes (56). Although WARS1 secretion has not been formally associated with IFN-γ in human macrophages, there is clear evidence of it being an IFN-γ target gene in other cells (57) and WARS1 expression levels have been shown to correlate with transcription factors involved in IFN-γ signaling such as *STAT1* (58). This also accords with the GSEA using MSigDB Hallmark that showed a significant downregulation of pro-inflammatory pathways in M1-like macrophages in response to fucoidan, in particular the interferon gamma and interferon alpha responses. The PPI network analysis further revealed a positive correlation between WARS1 and proteins of the interferon response, as well as a negative correlation with proteins involved in translation and tRNA aminoacylation.

Due to all the known effects of WARS1, its suppression as observed with fucoidan from *Saccharina latissma* may be an interesting anti-inflammatory strategy and thus one of the anti-inflammatory modes of action of fucoidan. Cytokine release experiments indeed confirmed that the native fucoidan from *Saccharina latissma* as well as degraded fractions with up to 100 times lower MW caused significantly reduced secretion levels of interferon gamma-induced protein 10 (IP-10/CXCL10), macrophage migration inhibitory factor (MIF), and urokinase-type plasminogen activator receptor (uPAR), all of which are known as IFN-γ inducible pro-inflammatory cytokines (59, 60). The rather unexpected finding that fucoidan from *Saccharina latissma* reduced both the WARS1 expression and the release of cytokines interferon gamma-induced protein 10 (IP-10/CXCL10) and urokinase-type plasminogen activator receptor (uPAR) seem to be independent of its MW is important, whereas macrophage migration inhibitory factor (MIF) was stronger effected in response to lower MW fucoidan.

Among the reduced cytokines, uPAR is of particular interest, since it has recently been recognized as a key regulator of inflammation. Initially, uPAR was considered primarily as a thrombolytic factor, whereas recent investigations revealed its predominant immunomodulatory functions in inflammation and cancer. uPAR is expressed as a glycophosphatidylinositol (GPI)-linked receptor and modulates adhesion and migration of different cell types, such as macrophages (61). Moreover, uPAR and soluble uPAR fragments (suPAR) are increased in viral sepsis (COVID-19), inflammatory bowel disease, and fibrotic diseases and are therefore suggested as a promising biomarker molecule for inflammatory diseases and cancer (61–63). Taken together, our study provides novel insights into the anti-inflammatory activities of fucose-containing sulfated polysaccharides from marine brown algae. However, the reduction in WARS1 expression and the reduction in downstream cytokine secretion are both observed in response to fucoidan, but it has not yet been demonstrated whether WARS1 suppression is causally responsible for the reduced cytokine release. These findings need further validation in additional *in vitro* and *in vivo* studies as well as dose-response experiments for native and degraded fractions in view of the underlying molecular mechanisms and potential medical applications.

## Data Availability

The data that support the findings of this study are available from the corresponding author (RP) upon reasonable request. The mass spectrometry proteomics data have been deposited to the ProteomeXchange Consortium via the PRIDE partner repository with the dataset identifier PXD082514.
